# Higher Risk of Intervertebral Disc Herniation among Neurosurgeons Than Neurologists: 15 Year-Follow-Up of a Physician Cohort

**DOI:** 10.3390/jcm7080198

**Published:** 2018-08-02

**Authors:** Wen-Cheng Huang, Chao-Hung Kuo, Jau-Ching Wu, Yu-Chun Chen

**Affiliations:** 1 Department of Neurosurgery, Neurological Institute, Taipei Veterans’ General Hospital, Taipei 11217, Taiwan; wchuang@vghtpe.gov.tw (W.-C.H.); chkuo6@vghtpe.gov.tw (C.-H.K.); 2 School of Medicine, National Yang-Ming University, Taipei 11221, Taiwan; 3 Department of Biomedical Engineering, School of Biomedical Science and Engineering, National Yang-Ming University, Taipei 11221, Taiwan; 4 Department of Family Medicine, Taipei Veterans’ General Hospital, Taipei 11217, Taiwan; 5 Institute of Hospital and Health Care Administration, National Yang-Ming University, Taipei 11221, Taiwan

**Keywords:** neurologists, neurosurgeons, intervertebral disc herniation, incidences, physician cohort, cohort study

## Abstract

High physical activity or workload has been associated with intervertebral disc degeneration. However, there is little data on physicians’ risks of disc disease. The study aimed to investigate the incidences of spinal problems among neurologists and neurosurgeons. A cohort of neurologists and neurosurgeons was derived from Taiwan’s national research database. During the study period, the incidences of intervertebral disc herniation or spondylosis among these specialists were calculated. Another one-to-one by propensity score matched cohort, composed of neurologists and neurosurgeons, was also analyzed. A Cox regression hazard ratio (HR) model and Kaplan-Meier analysis were conducted to compare the risks and incidences. The entire cohort comprised 481 and 317 newly board-certified neurologists and neurosurgeons, respectively. During the 15 years of follow-up, neurosurgeons were approximately six-fold more likely to develop disc problems than neurologists (crude HR = 5.98 and adjusted HR = 6.08, both *p* < 0.05). In the one-to-one propensity-score matched cohort (317 neurologists versus 317 neurosurgeons), there were even higher risks among neurosurgeons than neurologists (crude HR = 8.15, and adjusted HR = 10.14, both *p* < 0.05). Neurosurgeons have a higher chance of intervertebral disc disorders than neurologists. This is potentially an occupational risk that warrants further investigation.

## 1. Introduction

Herniation of intervertebral discs could cause back pain, radicular pain, or even muscle weakness. These symptoms have been demonstrated to cause a lower quality of life in patients when compared with healthy populations [1,2]. For example, herniated discs may cause significant morbidity and affect professional performance in athletes [3]. Studies have associated physical activity and workload with an increased risk of intervertebral disc herniation and degeneration [3,4,5,6,7,8,9]. There have also been reports on the association between occupation and risk of herniated intervertebral disc diseases. The results revealed that people frequently exposed to strenuous physical activity at work had a higher risk of herniated intervertebral disc, even without back pain history [10]. Furthermore, the risk of disc herniation increases when physical activity is performed improperly, and postural hygiene could mitigate this risk. Considering the long working hours of professional medical workers and the possibility of inappropriate postures during patient management, a study from Taiwan has already demonstrated that physicians might have a higher risk for lumbar herniated intervertebral disc than the general population [11]. However, there are scarce data on the incidences of such spinal problems among physicians or whether the disc diseases are considered as an occupational risk. Moreover, there has not been any study addressing the discrepancy of the incidences and risks among neurologists and neurosurgeons, who have distinct working styles while dealing with, in theory, similar patients.

The present study aimed to investigate the incidences of intervertebral disc herniation among professional medical doctors treating neurological disorders, who frequently encounter patients with the same problem. Furthermore, the study aimed to address the discrepancy in risks among neurologists and neurosurgeons, who treat the disorder differently (medical versus surgical, neurologists versus neurosurgeons, respectively). The present study uniquely acquired a cohort of physicians and surgeons over 15 years, using the National Health Insurance Research Database (NHIRD) of Taiwan. Due to the comprehensive and monopolistic coverage of Taiwan’s national health insurance [12], this cohort study followed-up every physician rigorously. Furthermore, other risk factors for spinal degenerative disc disease, for example, age and gender, were matched for comparison in the study. The workload of these neurologists and neurosurgeons was also estimated using their in-patient volume, which was precisely recorded in the NHIRD. To date, this is the first study to investigate the incidence rate of disc herniation among doctors and compare the risks among different medical specialties.

## 2. Experimental Section

### 2.1. Data Source and Ethical Concerns

The study used the NHIRD, which contains comprehensive information on every health care worker and insured subject of Taiwan, provided by the National Health Research Institute for academic use. The NHIRD allows detailed information of every individual (coded for deidentification), including gender, date of birth, date of medical board certification, residential or work area, dates of clinical visits, hospitalization, diagnoses, procedures, prescriptions, and expenditure amounts, etc. The diagnoses and procedures were recorded according to the International Classification of Diseases, 9th Revision, Clinical Modification (ICD-9-CM) codes and were originally used for billing. Thus, these data were internally monitored and cross-checked. Furthermore, the government-operated health insurance program, National Health Insurance (NHI) of Taiwan, has enrolled 99% of the population and provides unrestricted access to medical care in contracted clinics and institutions, which comprise 97% of the providers of health care services in Taiwan. Therefore, the NHIRD uniquely collected all data of patients, physicians, and physician-patients, and the cohort study had an extremely high rate of follow-up, theoretically almost complete follow-up of these physicians.

This study was approved by the Institutional Review Board of Taipei Veterans’ General Hospital, Taiwan (VGHIRB No.: 2012-10-008BC) and National Yang-Ming University Hospital (IRB No. 2015A015). Written informed consent from each of the enrolled was waived because all identifying personal information in NHIRD was stripped before analysis.

### 2.2. Study Cohorts

The study contained two cohorts, the original cohort, and the matched-cohort; both were extracted from the NHRID by identifying board-certified medical specialists.

In the original cohort, every newly certified neurologist and neurosurgeon was identified and included for follow-up. Exclusion criteria were previous history of spinal problems before the board-certification, unregistered physicians who had not been in practice in any of the hospitals, or whose certification was suspended.

The other matched cohort, a one-to-one matched comparison among neurologists and neurosurgeons, was derived from the original cohort based on a propensity score calculated by sex, level of hospital in which they were working, and in-patient service volume. The matched cohort, therefore, shared the same inclusion/exclusion criteria with the original cohort. Sex, age, hospital level, and service volume were considered covariates of the study.

**Figure 1:**
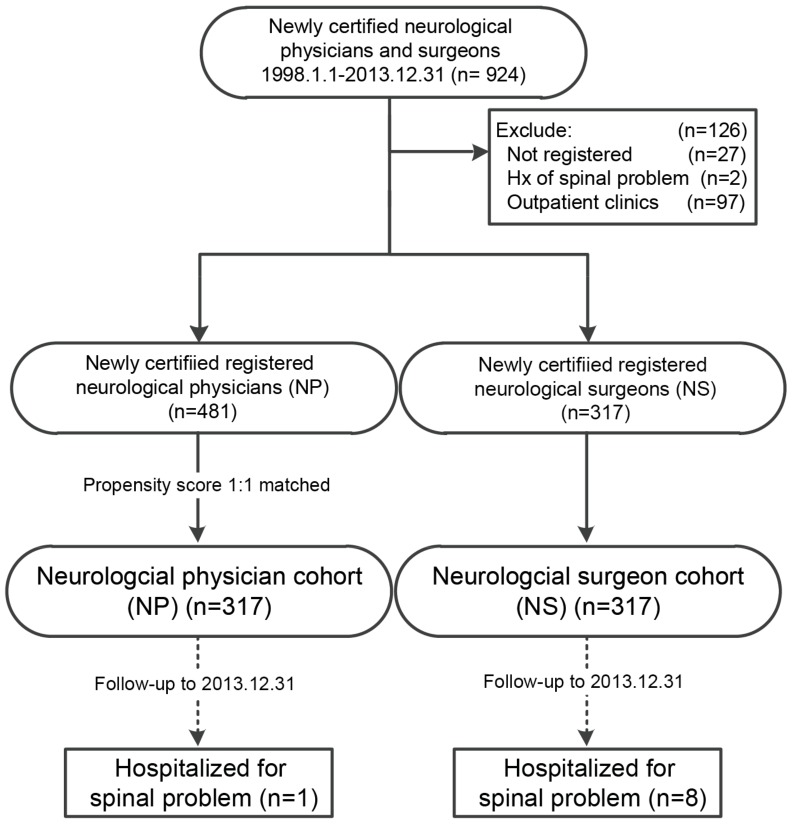
Flowchart of data processing for neurological physician cohort in Taiwan, 1998–2013. (*n* = 924).

Every neurologist and neurosurgeon was subsequently followed-up after their board certification. During the observation period, any hospitalization coded with the ICD-9 diagnosis of spinal intervertebral disc diseases (ICD-9-CM 721.×, 722.×, 723.×, and 724.×) was extracted and deemed as the study end-point. This cohort only censored follow-ups in the following conditions: the subject stopped practice (i.e., unregistered), expired, the date of the end-point incidence, or the end of this study, 31 December 2013 (Figure 1).

### 2.3. Hospital Level

All the medical specialists in Taiwan, including physicians and surgeons, are only allowed to practice in one hospital (to which they are registered). In the present study, each of the neurologists and neurosurgeons in Taiwan was assigned to the hospital where he or she received their board certification. These hospitals of Taiwan are evaluated regularly for hospital accreditation by the Joint Commission of Taiwan (http://www.jct.org.tw/FrontStage/doha_en.html), a government-sponsored organization, and classified into three levels: medical center, regional hospital, and district hospital. The highest level of hospitals, i.e., medical centers, in Taiwan, hold the highest standard in academics, quality of care, and responsibility of public health. Although the public might consider that the higher accreditation implies a better quality of medical services, the severity of diseases of the patients does not necessarily correlate with the classification of hospitals. However, the level of hospital, indeed, reflects some working styles of these medical specialists. For example, a larger proportion of medical centers are affiliated with medical schools and employ more university faculties. On the other hand, all these medical centers, regional hospitals, and district hospitals are contracted with the NHI program, which covers almost the entire population of Taiwan. Therefore, all the doctors, doctor-patient, and end-point events of the study were included in this cohort, no matter which hospital they went to or in which they worked in.

### 2.4. Service Volume of Physicians

Estimation of each neurologist’s and neurosurgeon’s workload was based upon the in-patient service volume. All physicians were classified into three approximate quantiles based on the annual volume of their in-patients, and ranked by neurologist and neurosurgeon, respectively. The cut off points were defined proactively to avoid potential bias resulting from ad-hoc analysis. Since the service volume could be subject to changes due to each physician’s occupational life, the rank of the last year of follow-up of each physician was used for analysis. The service volumes of these physicians were accordingly divided into three equal quantiles: low, medium and high.

### 2.5. Statistical Analysis

All of the data were linked using the SQL server 2017 (Microsoft Corp., Redmond, WA, USA) and analyzed by the SPSS software (SPSS, Inc., Chicago, IL, USA). The cumulative incidence rates of the study’s outcome (i.e., spinal disc diseases) were estimated and compared using the Kaplan-Meier method and Log-rank test. The Cox proportional hazard model was used to compare the hazard ratios of such outcomes among neurologists and neurosurgeons after adjustment for the covariates, including sex, age, hospital level, and service volume. A two-tailed level of 0.05 was considered statistically significant.

## 3. Results

Since 1 January 1998, there were a total of 924 newly board-certified neurologists and neurosurgeons identified in the NHIRD. After exclusion of those who had a history of spinal diseases (*n* = 2), not registered to practice (*n* = 27), or who never worked in a hospital (*n* = 97), there were 798 medical specialists in the original cohort.

The original cohort followed-up 481 neurologists and 317 neurosurgeons for up to 15 years (31 December 2013). The neurosurgeons were significantly more male predominant and older than the neurologists (both *p* < 0.001). The neurologists and neurosurgeons also had a different composition of the hospital level they worked in, and their volume of in-patient services (both *p* < 0.001) differed. The overall incidence rate of spinal disc diseases requiring hospitalization was 1.76 per 1000 person-years (Table 1). Neurosurgeons had a significantly higher chance of such a problem than neurologists (3.51 vs. 0.59 per 1000 person-years, crude hazard ratio (HR) = 5.98, *p* < 0.001). After adjustments made for age, sex, hospital level, and service volume, the neurosurgeons were six-fold more likely to have such problems (adjusted HR = 6.08, *p* < 0.05) (Table 2).

In the other matched (one-to-one by propensity score) cohort, composed of 317 neurologists and 317 neurosurgeons, the composition of gender, level of working hospital, and in-patient service volume were very similar, except the neurosurgeons were approximately 2.4 years older at the time of board-certification than the neurologists (Table 1). The risk of spinal disc problems was also significantly higher among neurosurgeons than neurologists (crude HR = 8.15 and adjusted HR = 10.14, *p* < 0.001 and *p* < 0.05, respectively) (Table 2).

**Figure 2:**
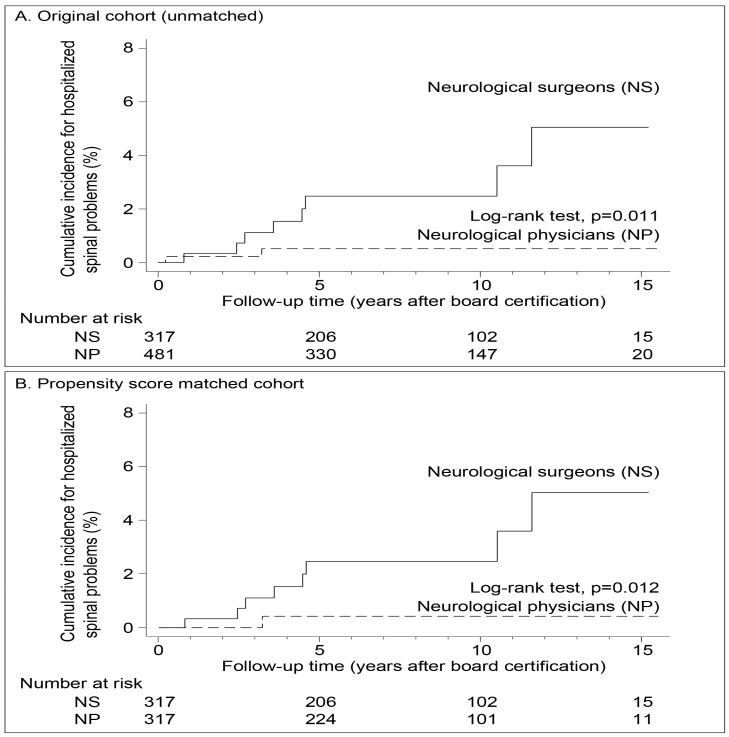
The Kaplan-Meier plot and Log-rank test for the incidence rates of spinal problems in NP and NS in the original cohort and propensity score matched cohort (1998–2013).

In the Kaplan-Meier analysis, the cumulative incidence rates of these hospitalized spinal problems were significantly higher among neurosurgeons than neurologists in both the original and matched cohorts (both *p* < 0.05) during the 15 years (Figure 2).

## 4. Discussion

This retrospective cohort study analyzed a total of 798 neurologists and neurosurgeons who worked in the hospitals of Taiwan. During the observation period of 15 years, the neurosurgeons were 6- to 10-fold more likely to develop spinal disc problems that required hospitalization than the neurologists. To date, there have been no other reports that have demonstrated the differential incidences and risks of spinal disc disease among medical specialists. Using the NHIRD, which comprehensively covers health care providers and consumers of Taiwan, the specific cohort of medical specialists had little selection bias. The study completely followed-up every doctor enrolled. This was the first study that focused on neurologists’ and neurosurgeons’ risks of spinal disc diseases, which could be correlated with the differences in their working style and workload. The results could imply the occupational risk of spinal disc problems among medical professionals. Although the incidences and risks were not high, the remarkable difference among the two specialties, neurology and neurosurgery, treating, presumably, a similar group of patients, warranted notice and further investigation.

Intervertebral disc herniation and spondylosis are diseases of a degenerative nature and, thus, are intuitively associated with wear and load. It is commonly accepted that older age, obesity, and high physical activity are risk factors of spinal disc protrusion [13,14,15]. There are also a few reports addressing certain jobs and activities, including football and heavy lifting, that may predispose subjects to such problems [3,4,9,15,16,17]. The mechanism of spinal disc herniation is generally correlated with an excessive load over the vertebral segment and deficiency of structural strength, for instance, a torn annulus fibrosis. Therefore, lifting heavy loads repeatedly, non-ergonomic postures for a prolonged time, and lengthy working hours could accelerate degeneration and protrusion of intervertebral discs. In a nationwide population-based study in Taiwan, Chan et al. illustrated the risk of lumbar herniated intervertebral discs among physicians, non-physician health care professionals, and the general population. The results revealed that physicians and non-physician health care professionals had a higher L-HIVD risk than the general population, but there was no difference between physicians and non-physician health care professionals [11]. It is also reasonable to infer that neurosurgeons encounter more of the aforementioned situations than neurologists during work, since neurosurgical operations often take many hours. The neurologists, presumably, can better adapt posture, ergonomics and hours of work better than neurosurgeons due to more flexible working schedules. It was the authors’ theory that neurologists, thus, might have a lower risk of spinal disc problems than neurosurgeons. The study deliberately chose a cohort composed of neurologists and neurosurgeons, whose clinical work, theoretically, covered patients of substantial similarity, for comparison.

There are also several other risk factors for intervertebral disc herniation, such as obesity, hyperlipidemia, and cardiovascular diseases [6,8,18,19]. Interestingly the aforementioned three conditions frequently coexist among patients of spondylosis and disc herniation. Moreover, cigarette smoking has been reported to be correlated with such spinal disc disorders [20,21]. Since these risk factors frequently overlap and can predispose one another, they are unlikely to be independent for disc herniation. Although the contribution of each is still uncertain, their interaction and influences are worth further investigation. These factors were not addressed in the current study. However, given that the comparison was made between doctors of two different medical specialties, neurology and neurosurgery, there should be little selection bias. The two groups of doctors probably shared similar co-morbidities of obesity, hyperlipidemia, and cardiovascular disease. On the other hand, only a few studies have investigated the risk of spinal problems and other musculoskeletal disorders among different medical specialties [22,23,24]. One study which enrolled 94 cardiologists demonstrated that interventional electrophysiologists had a higher prevalence of spondylosis than non-interventional cardiologists. The results indicated that the prolonged wearing of lead during the fluoroscopic procedures likely posed extra loading to the operator’s back, and, thus, caused the disc herniation. The findings were compatible with the theory that excessive loading for prolonged periods, for example, the suboptimal ergonomics for the operator during a neurosurgical operation, could potentially damage the intervertebral discs.

There are limitations to the current study. It was difficult to precisely localize the level of disc herniation of each physician-patient. The NHIRD kept precise records of the diagnostic codes of each hospitalization and they were initially meant for billing purpose, so it was not possible to trace each patient’s radiological reports or operative notes to analyze the exact level or size of, and neurological dysfunction caused by, the herniated nucleus pulposus. It was also difficult to investigate each subject’s body mass index, cigarette consumption status, or exact workload. For similar reasons, there were no records of the working hours of the neurologists and neurosurgeons, such as the amount of time spent in the clinics or operating theaters, which could accurately reflect the occupational exposure. On the other hand, the study used the volume of in-patients of each neurologist and neurosurgeon to estimate their workload. To ameliorate the confounders of each group, the scale of the hospital that these medical specialists were working in was also categorized into three levels for comparison. Furthermore, despite the large cohort of 798 medical specialists retrospectively followed-up, the incidence rates of an outcome event—hospitalization for disc herniation—were still low. The study power could be enhanced further by increasing the sample population. A larger sample size would be required to validate the risk of spinal disc problems demonstrated in the cohort study. Nevertheless, the outcome reported in the current cohort was all hospitalizations for disc herniation, which could be considered as a definite consequence of such a disease. The outcome events would not have included those moderate spondyloses or bulging discs of minor symptoms or equivocal diagnosis. Thus, the incidence rates reported were likely an under-estimation. The true risks of the spinal disc disease could be even higher.

This retrospective cohort merits the comprehensiveness of inclusion of a generation of newly board-certified neurologists and neurosurgeons with the nearly-perfect follow-up during the observation period. It is likely that the data for the development of spinal disc problems in these physician-patients who required hospitalization were all captured. By control of other covariates (i.e., age, sex, hospital scale, and service volume) the differences in the outcome event demonstrated among neurologists and neurosurgeons warrant further attention. This is potentially a risk that might adversely affect a specialist’s career and could be avoided or at least reduced.

## 5. Conclusions

Higher incidences of intervertebral disc disorders were demonstrated in neurosurgeons than neurologists. Whether this is potentially an occupational risk warrants further investigation.

## Figures and Tables

**Table 1:** Demographic features between neurological physicians (NP) and surgeons (NS) in original cohort and propensity score matched cohort (1998–2013).

Demographic Features	Original Cohort					Propensity Score Matched Cohort ^1^				
	Neurological Physician, NP		Neurological Surgeons, NS			Neurological Physicians, NP		Neurological Surgeons, NS		
	*n* = 481	(%)	*n* = 317	(%)	*p*-Value	*n* = 317	(%)	*n* = 317	(%)	*p*-Value
Gender					<0.001					1.000
Male	329	(68.4)	307	(96.8)		307	(96.8)	307	(96.8)	
Female	152	(31.6)	10	(3.2)		10	(3.2)	10	(3.2)	
Age of board certification (mean ± SD)	31.5	±3.5	34.2	±2.4	<0.001	31.8	±3.4	34.2	±2.4	<0.001
Working hospital level					<0.001					0.061
Academic medical centers	185	(38.5)	135	(42.6)		112	(35.3)	135	(42.6)	
Metropolitan and local hospitals	296	(61.5)	182	(57.4)		205	(64.7)	182	(57.4)	
Physician service volume					<0.001					0.648
High	223	(46.4)	123	(38.8)		126	(39.7)	123	(38.8)	
Middle	126	(26.2)	99	(31.2)		90	(28.4)	99	(31.2)	
Low	132	(27.4)	95	(30.0)		101	(31.9)	95	(30.0)	
Outcome										
Hospitalized for spinal problem	2	(0.4)	8	(2.5)	0.017	1	(0.3)	8	(2.5)	0.038

^1^ Propensity scores were calculated using multiple logistic regression with sex, working hospital level, and physician service volume.

**Table 2:** Incidence rates and hazard ratios for spinal problems in NP and NS in original cohort and propensity score matched cohort (1998–2013).

Admission for Spinal Problem	Original Cohort				Propensity Score Matched Cohort ^1^			
	Total	NP	NS		Total	NP	NS	
Incidence (per 1000 person-years)	1.76	0.59	3.51		1.95	0.43	3.51	
Number of occurrences	10	2	8		9	1	8	
Observed person-years	5692.3	3411.0	2281.3		4606.6	2325.3	2281.3	
Crude HR (95% C.I.)		1.00	5.98	(1.19–57.81) ***^,2^		1.00	8.15	(1.19–361.84) *^,2^
Adjusted HR (95% C.I.)		1.00	6.08	(1.15–32.12) *^,2^		1.00	10.14	(1.14–90.22) *^,2^

^1^ Propensity scores were calculated using multiple logistic regression with sex, working hospital level, and physician service volume. ^2^ Statistical significances: * *p* < 0.05; *** *p* < 0.001.
